# Lysergic Acid Diethylamide (LSD) and the Heart: Exploring the Potential Impacts of LSD on Cardiovascular Function

**DOI:** 10.7759/cureus.87356

**Published:** 2025-07-05

**Authors:** Akshita Suleria, Sakshi Verma, Khanij Arya, Mason T Stoltzfus, Ira Gupta, Bhupinder Singh, Tanveer Shaik

**Affiliations:** 1 Psychiatry, Government Medical College, Patiala, Patiala, IND; 2 Internal Medicine, Government Medical College, Amritsar, Amritsar, IND; 3 Neurosurgery, Penn State College of Medicine, Hershey, USA; 4 Internal Medicine, Icahn School of Medicine at Mount Sinai, Queens Hospital Center, Queens, USA; 5 Medicine, Avalon University School of Medicine, Willemstad, CUW

**Keywords:** cardiovascular disease, lysergic acid diethylamide, microdosing, psychedelics, serotonin

## Abstract

Lysergic acid diethylamide (LSD) is an ergot-derived psychedelic agent that produces perceptual and psychic effects of heightened sensations by acting on the dopaminergic, adrenergic, and serotonergic pathways in the brain and periphery, with 5-hydroxytryptamine 2A (5-HT2A) as the primary target molecule. Its action on these receptors in the central nervous system is comparatively well studied with respect to the psychedelic effects; however, there is speculative evidence of cardioprotective effects in the current literature attributed to the usage of this substance, even though acute ingestion causes tachycardia and hypertension, just like other psychedelics. Larger recreational doses of the drug can lead to cardiovascular and cerebrovascular incidents, but chronic peripheral antagonism of 5-HT2A receptors by the drug reduces atherosclerotic and thrombotic processes due to a reduction in platelet aggregation and vascular smooth muscle cell proliferation. Central sympathetic stimulation caused by micro-dosing of LSD imparts anti-inflammatory effects and increases cortical synaptogenesis, leading to reduced chronic inflammation implicated in causing cardiovascular diseases.

## Introduction and background

Psychedelics are drugs that alter the mind and state of consciousness due to psychoactive properties like hallucination and dissociation. They are mainly classified into tryptamines, lysergamides, phenethylamines, cannabinoids, and atypical psychedelics [1,2]. Lysergic acid diethylamide (LSD) is the most notable psychedelic belonging to the class of lysergamides. It was initially synthesized using ergotamine or ergotamine tartrate as starting materials in covert labs and was heavily misused for its psychedelic effects throughout the world in the 1960s [3]. A sufficiently high dose of LSD is found to cause visual hallucinations and delusions that alter the user's perception of time and self [4].

In the United States, 27 million people aged 12 and older reported using LSD in the last year, according to the 2018 National Survey on Drug Use and Health (NSDUH) [5]. The Drug Abuse Warning Network (DAWN) states LSD was responsible for 4,819 emergency department visits in 2011 [5]. LSD is taken orally in tablets (microdots), gelatine squares (window panes), and impregnated sugar cubes. It is sold in a variety of formulations with effects that start appearing within 30-60 minutes after consumption and persist for 10-12 hours [5]. The lethal dosage (LD50) of LSD ranges from 200 μg/kg to more than 1 mg/kg of human body mass, while most sources state that no such overdose has been reported in humans [6]. On ingestion, LSD heightens sensory awareness with an increased sense of clarity accompanied by a decreased perception capacity. The user may experience perceptual alterations, including visual distortion in the size, motions, color, sound, touch, and forms of surrounding objects and the user's body image. Users experience perceptual, emotional, and systemic effects, including altered time perception, mood swings, and cardiovascular changes [2,5].

Cardiovascular disturbances that occur in an ambiguous and unexplained fashion should be suspected to be caused due to illegitimate drug ingestion [7]. These disturbances could include tachycardia and/or hypertension. In some instances, supraventricular tachyarrhythmias and myocardial infarction have been documented. Studies have shown that if controlled care is given while classic psychedelics are consumed, psychological well-being is enhanced, and risk factors for cardiometabolic diseases are reduced. These risk factors include unhealthy diet, alcohol, and tobacco consumption, leading to a poor lifestyle [2]. However, much of this evidence comes from observational studies or self-reports and may reflect indirect behavioral changes rather than direct drug effects. Recent randomized trials have shown that assisted therapy using LSD in a psychotherapeutic setting has anxiolytic effects, producing long-lasting curtailment in anxiety and comorbid symptoms of depression [8,9]. Some have speculated that such benefits may extend to cardiovascular health through stress reduction or inflammatory modulation, but these remain unconfirmed.

This review does not assume therapeutic benefit but rather aims to explore the mechanisms by which LSD may influence cardiovascular health by exploring underlying mechanisms and comparing the existing literature, as this topic is of increasing importance given that there are significant efforts underway to commercialize psychedelics as medicines [10].

## Review

Pathophysiology

**Figure 1:**
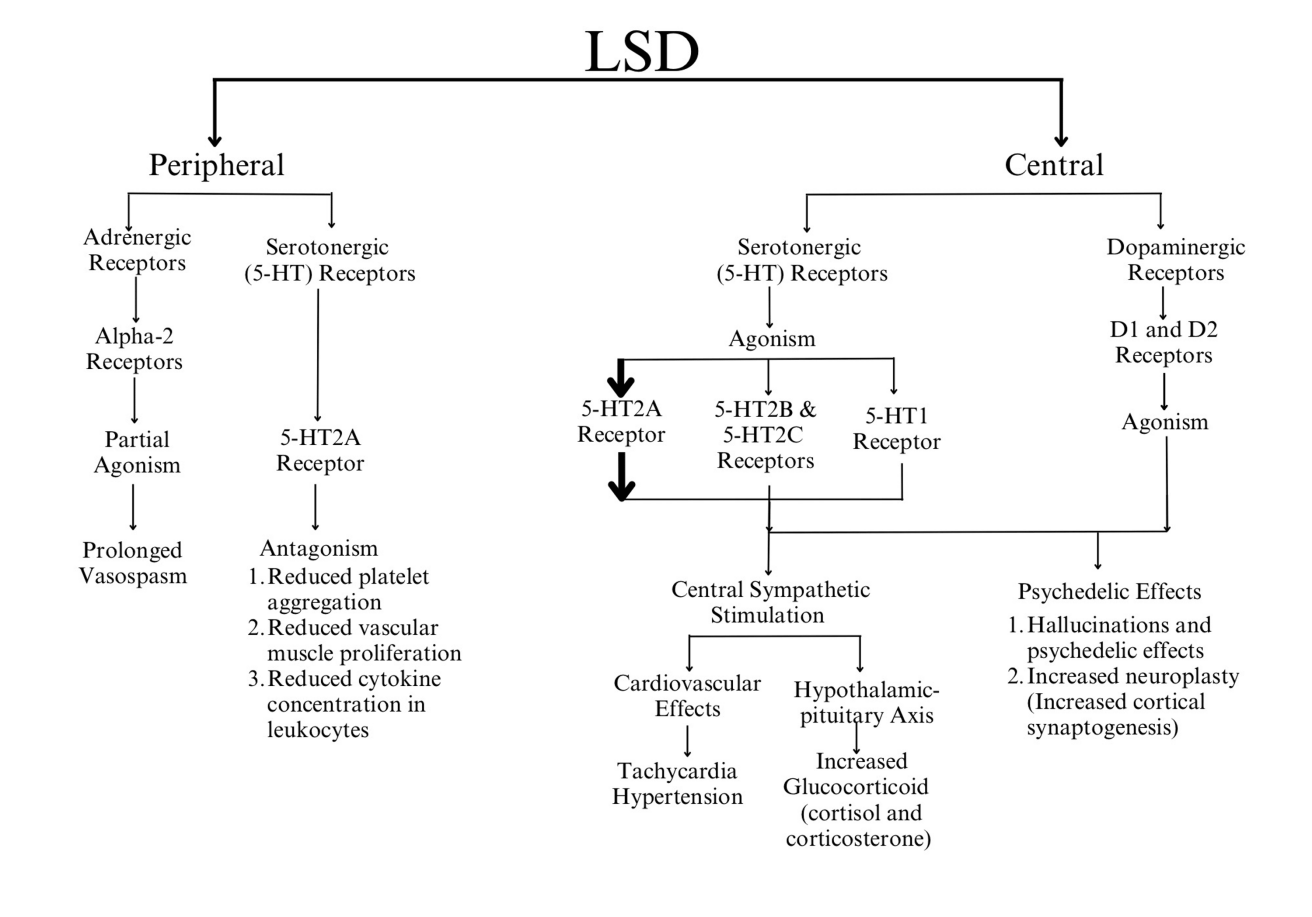
Peripheral and central effects of LSD Image Credit: Authors. LSD: lysergic acid diethylamide; 5-HT: 5-hydroxytryptamine; D1: dopamine 1; D2: dopamine 2

LSD is absorbed from the gastrointestinal tract, and its actions start in 30 minutes, lasting for 6-12 hours [11]. It acts on the serotonergic, dopaminergic, and adrenergic receptors in both the periphery and the central nervous system (CNS) (Figure 1).

Psychedelic effects are primarily attributed to the agonism of serotonin (5-hydroxytryptamine (5-HT)) via 5-HT2 and 5-HT1 autoreceptors in the brain after crossing the blood-brain barrier. Activation of 5-HT2A receptors in the neocortical pyramidal cells and the hippocampal prefrontal cortex is the cause of heightened sensory stimuli and hallucinations associated with LSD [12,13]. Apart from this, LSD also acts on the central dopaminergic receptors D1 and D2, where D2 activation is known to potentiate 5-HT2A effects; thus, an interplay between central dopaminergic and serotonergic pathways results in the diverse range of psychedelic actions of this drug [14].

Actions of LSD on the cardiovascular system are primarily due to stimulation of the central sympathetic system via activation of 5-HT2A receptors, which causes tachycardia, rise in blood pressure, mydriasis, and hyperthermia [14]. Cardiovascular effects of LSD vary with its usage and hence can be broadly classified as chronic and acute.

Chronic effects due to serotonergic interaction at the periphery

5-HT2A Receptor and Platelet Aggregation

5-HT is a monoamine released from platelets and mast cells, which also acts as an important neurotransmitter in the CNS. 5-HT receptors are classified into different types based on their location and function. LSD acts as a partial agonist on the 5-HT2, 5-HT1, and 5-HT5 receptors at various sites [11,15]. Its primary action is on 5-HT2A receptors in the cerebral cortex, platelets, vascular smooth muscle cells, skeletal muscle cells, and the kidney. The drug is known to show agonism in the CNS while antagonism in the periphery in platelets and vascular smooth muscle cells [11,16].

**Figure 2:**
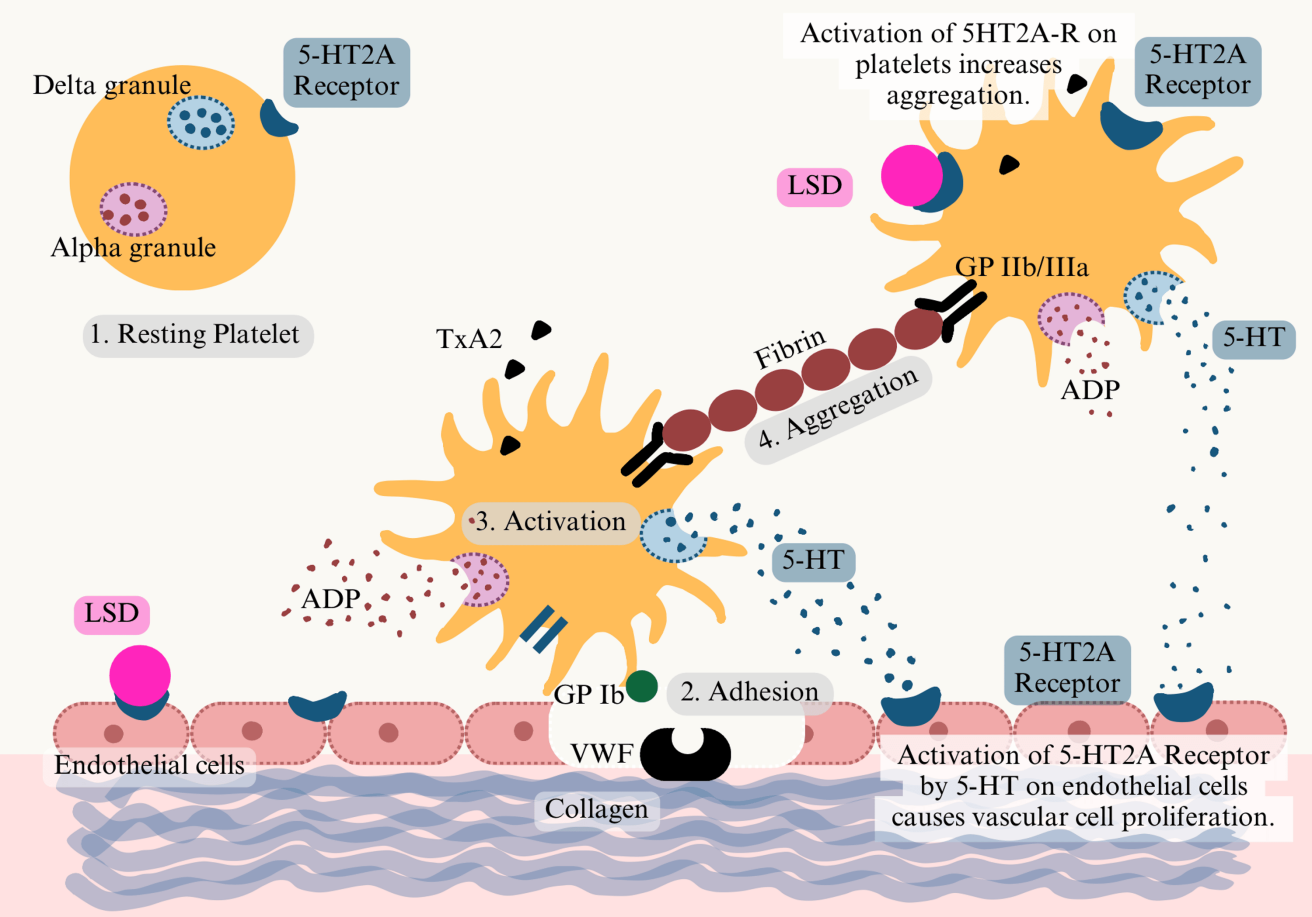
Effect of LSD on platelet function Image Credit: Authors LSD: lysergic acid diethylamide; 5-HT: 5-hydroxytryptamine (seratonin); VWF: Von Willibrand factor; GP Ib: glycoprotien Ib

Hemostasis relies on a complex interaction between platelets, vascular endothelium, and clotting factors to stop bleeding at the injury site, where platelet activation and aggregation form the basis of primary hemostasis, i.e., platelet plug formation. Endothelial injury exposes collagen and Von Willebrand factor (VWF), which bridges platelet adhesion with the endothelium via glycoprotein IB (GPIB) receptors. This adhesion induces platelet activation, leading to a conformational change in the platelet shape with the subsequent release of cellular contents, including 5-HT, thromboxane A2, and adenosine diphosphate (ADP) [17]. 5-HT2A activation increases intracellular calcium, promoting platelet aggregation and smooth muscle proliferation via PLC, PKC, and DAG/IP3 pathways [16,18,19]. However, receptor activation also directly releases arachidonic acid and 2-arachidonoylglycerol, indicating three distinct biochemical pathways on receptor activation [20]. Thus, activation of the 5-HT2A receptor is a key step in platelet aggregation and smooth muscle proliferation. Abnormal activation of this pathway because of chronic inflammation and lipid abnormalities leads to endothelial dysfunction and aids in atherosclerosis and thrombosis, linked to cardiovascular morbidity. Since LSD acts as an antagonist on this receptor in the periphery, it could theoretically reduce atherosclerotic events by altering platelet aggregation pathways (Figure 2) [11].

The first clinical association of increased serotonergic activity with valvular heart disease (VHD) was observed in patients with carcinoid syndrome, where chronic 5-HT excess led to right-sided valve fibrosis. This effect is mediated via activation of the 5-HT2B receptor, which stimulates myofibroblast proliferation and glycosaminoglycan deposition on heart valves, progressing to fibrosis, regurgitation, arrhythmias, and eventually heart failure [21].

Several ergot-derived agents, such as methysergide, ergotamine, pergolide, and dihydroergotamine (DHE), are known to cause retroperitoneal, pleural, and cardiac fibrosis, primarily via potent 5-HT2B receptor agonism. Notably, LSD, which shares structural similarity with these compounds, has a Ki value of 0.98 nM at the 5-HT2B receptor, which is well below the 15 nM threshold commonly associated with valvulopathy risk. Even a single dose of cabergoline, another high-affinity 5-HT2B agonist, has been reported to cause valvular pathology in humans [22].

Upon receptor activation, a cascade of pro-fibrotic signaling pathways is triggered, involving phospholipase C-β (PLC-β), protein kinase C (PKC), Src phosphorylation (Src-P), and transforming growth factor-beta 1 (TGF-β1). These pathways converge with extracellular signal-regulated kinase (ERK) and mitogen-activated protein kinase (MAPK) signaling, leading to fibroblast activation, extracellular matrix remodeling, and irreversible valvular fibrosis [22,23].

Although direct clinical evidence linking LSD use to VHD remains limited, the mechanistic threat is biologically plausible and increasingly concerning. LSD’s high-affinity binding to 5-HT2B receptors and its ergot-like structures strongly suggest a theoretical risk of valvulopathy, particularly with chronic microdosing protocols. However, whether in vivo plasma concentrations achieved through typical LSD use are sufficient to activate this fibrotic cascade remains unknown. Future studies must address this critical pharmacokinetic-pharmacodynamic gap by correlating LSD blood levels with the receptor occupancy threshold required to induce valvular fibrosis [23].

Interaction with the Immune System

LSD stimulates the central sympathetic system via 5-HT2 and 5-HT1 receptors, activating the HPA axis and raising cortisol and corticosterone levels [24-26], which have well-known immunosuppressive effects. These include reduced pro-inflammatory cytokines and dampened immune cell activity. Additionally, preclinical studies show 5-HT2A activation, as with the agonist, DOI, can acutely suppress tumor necrosis factor alpha (TNF-α), IL-6, IL-1beta, and adhesion molecules like ICAM-1 and VCAM-1 in animal models [27,28]. These findings suggest that LSD’s immunomodulatory effects may arise via two independent mechanisms: 5-HT-mediated cytokine suppression and HPA axis-driven glucocorticoid release. While distinct, these pathways may interact to influence chronic inflammation. However, these effects are not confirmed in humans, and clinical data on LSD’s impact on chronic inflammation are lacking. Any immunomodulatory action likely results from transient glucocorticoid effects and acute receptor activity, rather than sustained immune changes. Their combined influence on long-term cardiovascular risk remains theoretical.

Acute Effects Due to High-Dose Usage

Acute cardiovascular effects of LSD are a result of central sympathetic stimulation along with adrenergic activation, leading to systemic effects like tachycardia [14,15,29]. LSD is derived from ergot alkaloids, which are known to cause adrenergic receptor activation. These compounds act as partial agonists and dissociate slowly from the alpha receptors, causing prolonged vasospasm, which is partially reversed by alpha-blockers [11]. This prolonged vasospasm only happens with a huge recreational dose (>100 μg) of LSD [30]. It can cause major cardiovascular incidents like carotid artery occlusion and peripheral vascular occlusion, leading to cardiovascular collapse, cerebrovascular incidents, and peripheral ischemia in susceptible individuals [31].

Discussion

LSD is a psychedelic agent that has been recognized as a Schedule 1 controlled substance by the United Nations since 1971 [32]. Most of the literature on the drug's effects has been based on research before this time. The past two decades have witnessed increased work on identifying the mechanisms by which psychedelics work to identify speculated benefits of long-term usage on various systems in the human body.

Even though sparse, current literature shows heterogeneity regarding the effects of LSD usage on the cardiovascular system. Ingestion of LSD shows dose-dependent effects and hence can be classified broadly into acute effects due to high dosage and chronic effects due to frequent recreational doses. A cross-sectional study done by pooling and comparing data from the National Survey on Drug Use and Health (2005-2014) in the United States by Simonsson et al. in 2021 found self-reported reduced incidence of heart disease and diabetes in the past year in patients with LSD usage with the adjusted odds ratio (aOR) of 0.77, 95%CI: 0.65-0.92; p = 0.006 and aOR of 0.88, 95%CI: 0.78-0.99; p = 0.036 respectively, defining reduced odds of suffering from heart disease and diabetes in the past year by 23% and 12%, respectively, for the participants who ever used classic psychedelics [2]. However, it is important to note, when regression analysis was conducted to measure the strength of three different classes of classic psychedelics (tryptamine, LSD and phenethylamine), no class of classic psychedelics use was found to be significantly associated with heart disease and diabetes in the past year with only exception to tryptamine use being significantly associated with decreased odds of diabetes. This suggests that even though several confounders were controlled by the regression model, the associations still could have been affected by hidden confounders, for example, there could exist a common factor predisposing individuals to both psychedelic use and heart diseases. It is also important to note that the term ‘heart disease’ could include a wide range of diseases, and ‘diabetes’ could pertain to either or both type 1 and type 2 diabetes mellitus. Therefore, the association could vary across the different types of heart diseases and diabetes [2]. In a similar study by the same authors, Simonsson et al. assessed the incidence of hypertension with chronic psychedelics, including LSD usage [33]. Still, they concluded no significant association in preventing hypertension. Both of these studies could not accurately account for frequency, context of dose, and other confounding factors, but still speculated usage of the drug with reduced incidence of heart diseases, even though unspecified [2,33].

The main target molecule of LSD is a 5-HT autoreceptor, i.e., 5-HT2A. It has been studied in mouse model experiments that the activation of the peripheral 5-HT2A system, i.e., an increase in 5-HT synthesis, upregulated 5-HT2A receptor expression, and decreased 5-HT degradation, has a role in the pathogenesis of atherosclerosis. Antagonism of the 5-HT2A in these mouse models has confirmed atherosclerosis modulation by inhibiting the growth of smooth muscle cells and macrophage foam cells [34]. LSD acts as an antagonist on this receptor on platelets and smooth muscle cells, but there is a paucity of human evidence directly linking LSD to reduced atherosclerosis, even though the proposed mechanism plausibly supports the link between the leading site of action of LSD on the 5-HT2A receptor and its potential cardioprotective effects [16].

Szabo A et al. described the mechanisms through which LSD may exert anti-inflammatory effects by altering lymphocyte function [1]. In vitro exposure to high concentrations of LSD (100 µM) significantly inhibited cytokine proliferation, B cell activation, and NK cell responses; lower doses (0.0001 and 0.1 µM) augmented NK cell functions, though these effects may differ in vivo. These in vitro findings, often at supra-physiological concentrations, limit clinical applicability. Chronic inflammation and circulating cytokines like TNF-α contribute to atherosclerosis. Cloëz-Tayarani et al. found that selective 5-HT2A receptor activation reduced TNF-α in LPS-stimulated human peripheral blood mononuclear cells [27]. This was further supported by Nau et al. using the 5-HT2A receptor agonist 2,5-dimethoxy-4-iodoamphetamine (DOI), which reduced TNF-α levels in vivo [28]. 

However, these findings were challenged by Rudin et al., who noted that the concentrations of 5-HT and DOI (up to 100 μM) used in prior experiments exceeded normal human plasma levels, rendering the results clinically irrelevant. Their study showed that LSD, at typical doses, did not directly alter cytokine release or lymphocyte proliferation in human T-cells, suggesting it may be safely used in immunocompromised patients without affecting immune function [25]. Central sympathetic stimulation via 5-HT2 and 5-HT1 receptor agonism in the brain, leading to HPA axis and increased circulating glucocorticoids, may produce indirect anti-inflammatory effects, rather than direct immune modulation [20,26,35].

In a controlled research setting, a single dose of LSD does not produce any major adverse cardiovascular effects and is usually well tolerated when used in a 50-200 μg dose, with addiction potential only observed in poly-drug users [36]. In contrast, low-dose or micro-dosing of LSD has been presumed to have different neurological and psychological consequences when compared with the higher drug usage [37]. Micro-dosing refers to the usage of a lower than the usual recreational dose (5-15% of a normal recreational dose, i.e., 10 μg of LSD) over a longer period of time and has been speculated to provide behavioral benefits among the users. A study by Anderson et al. in 278 individuals with the history of microdose psychedelic use including LSD showed spontaneous improvements in meditative practice (49.1% of participants), exercise (49.1%), eating habits (36.0%) and sleep (28.8%); with reduced use of caffeine (44.2%), alcohol (42.3%) and tobacco (21.0%) presumed to occur due to modification in behavior as a result of neuroplastic action on promoting cortical synaptogenesis by the drug [38]. This behavior change can be attributed to the REBUS (relaxed beliefs under psychedelics) model, which states that psychedelic use alters brain entropy leading to a multilevel change in behavior leading to long-term changes like improvement in lifestyle choices, including better abstinence rates in alcoholics and tobacco users which could explain the reduced cardiovascular morbidity due to reduction in these risk factors [39,40].

Despite these documented benefits, LSD microdosing is found to have adverse effects, questioning its safety. It is also worthwhile to know that every benefit of microdosing could have the exact opposite adverse effects in some subjects. Anderson et al. in their study showed that if a microdose of LSD improved the mood in some, it also impaired the mood in others [38]. Hence, it becomes important to consider confounders like placebo effect and individual differences, which could lead to different effects in different people. In another double-blinded randomized control trial, theauthors investigated the effect of four repeated doses of LSD tartrate or placebo, given to healthy participants at intervals of three to four days, on mood and cognitive function [41]. Three different drug conditions were randomly allocated to participants: placebo (n=18), 13 μg LSD (n=19), or 26 μg LSD (n=19). After the intervention, LSD microdosing was found to have no effect on mood, psychomotor performance, or emotional tasks. The authors concluded that while repeated low doses of LSD are safe in a controlled environment with a limited number of administrations, they had no effect on the mood or cognitive abilities of healthy volunteers [41]. So, there are concerns, particularly with LSD microdosing, that expectancy may be driving claims of therapeutic efficacy in the community. 

Many recent studies have highlighted the impact of chronic LSD microdosing on the risk of VHD. Although, safety of continuous microdosing over the long term is not well established, activation of 5-HT2B receptors has been linked to a possible risk of VHD [23]. Pro-fibrotic pathways are thought to be activated by 5-HT2B receptor stimulation by chronic LSD microdosing through the activation of phospholipase C-β, protein kinase C, Src-P, and transforming growth factor-β1. Moreover, there could be a role for MAPK signalling. It has been demonstrated that activation of the ERK and p38 MAPK pathways can activate fibroblasts, and that MAPK signalling pathways can boost the activation of cardiac fibroblasts, resulting in increased production of extracellular matrix proteins [42]. It is also thought that an LSD microdose might be large enough to increase the risk of VHD, particularly when taken over a long period of time [22]. As emphasized by Tagen et al., the absence of well-designed animal or clinical studies creates a significant gap in our understanding and reinforces the need for future investigations to focus on defining the threshold concentration of LSD required to activate pro-fibrotic pathways and whether plasma levels achieved in typical therapeutic or recreational use approach that threshold [23]. Until such data are available, the valvular safety profile of LSD remains mechanistically worrisome and clinically unresolved, warranting precaution in its medical and experimental use.

LSD, being an ergot derivative, is known to cause prolonged vasospasm in certain individuals. Akasaki and Ohishi conducted a review on acute adverse effects of psychedelic use, which have shown large dosages of LSD to cause dangerous cardiovascular complications [31]. They described a case of LSD-induced vasospasm and gangrene formation in the patient's lower limb three days after using the drug, who was treated with transluminal angioplasty of the affected arteries and maintained well after that [43]. They also reviwed a case of a 20-year-old woman with high-grade stenosis at the level of the siphon of the internal carotid artery after ingestion of LSD, suggestive of vasoconstriction as the underlying mechanism [44]. A similar case of a previously healthy 14-year-old boy, after ingesting four capsules of LSD, presented with focal neurological deficits due to carotid artery obstruction, suggesting ergot alkaloid-like vasospastic action of LSD in patients with hypersensitive vessels [45]. Aakerøy et al. described a case of a young man with an unremarkable medical history who suffered a seizure with subsequent cardiorespiratory arrest and severe neurological sequelae after ingesting a blotter followed by supraventricular tachycardia with lactic acidosis suggesting severe cerebral hypoxia on ingestion of 300 μg of LSD which is way higher than the usual 20-30 μg of recreational dose [46]. These cases of lower extremity vasospasm, cardiovascular graft rejection due to vasospasm, seizure-induced supraventricular tachycardia, and carotid artery vasospasm in individuals with prior LSD use as the only significant history suggest the probable dangerous acute effects of the drug. These acute effects could be due to improper biosynthesis of the drug from ergot alkaloids or due to retained alpha-adrenergic action of LSD itself and need to be considered as possible outcomes of drug use in further research. Hence, while LSD may exhibit cardioprotective function in the long run, rare but known risks of vasospasm in susceptible individuals with acute large doses pose a challenge in identifying the actual benefits of the drug. Table 1 summarizes the potential cardiovascular effects and/or side effects of LSD as mentioned in various studies.

**Table 1:** Potential cardiovascular effects and/or side effects of LSD NSDUH: National Survey on Drug Use and Health; VHD: valvular heart disease; FAERS: FDA Adverse Event Reporting System; ERK: extracellular signal-regulated kinase; MAPK: mitogen-activated protein kinase; MDMA: 3, 4-methylenedioxy-N-methamphetamine; TNF-α: tumor necrosis factor alpha; PBMC: peripheral blood mononuclear cell; DOI: 2,5-dimethoxy-4-iodoamphetamine; CDP: cyclodextrin polymer; SH: sarpogrelate hydrochloride; LSD: lysergic acid diethylamide

Author, Year	Study type/participants	Cardiovascular and other described effects
Szabo, 2015 [1]	Review Article	Classical psychedelics not only affect the brain but also modulate immune responses by influencing key inflammatory and cell survival pathways through receptors like serotonin and sigma-1. This immunomodulatory potential suggests new therapeutic avenues for treating chronic inflammatory and autoimmune diseases, though the field remains underexplored.
Simonsson et al., 2021 [2]	Cross sectional study; 376,207 participants were analysed for past one year prevalence of heart disease in lifetime psychedelic users.	Survey on data pooled from NSDUH was utilized to find association between lifetime psychedelic (LSD) use and prevalence of heart diseases. Even though the results showed lower odds (aOR = 0.77 (95%CI 0.65-0.92) p=0.006) of heart disease in the past one year in participants, the range of potential confounding factors, specific dosage frequency and type of heart disease was not accounted for. Moreover, LSD use specifically was associated with an aOR of 0.88 (95%CI 0.73-1.07) and p = 0.199 which does not signify significant association.
Raymond et al., 2001 [16]	Review Article	Focused on the signaling mechanisms of G-protein-coupled serotonin (5-HT) receptors. Summarizes current knowledge on the classification and diversity of 5-HT receptors—highlighting how gene variants and post-genomic modifications expand the functional complexity to at least 30 distinct receptors—and discusses recent findings in their signaling pathways.
Urban et al., 2007 [20]	Experimental pharmacological study using in vitro cell-based assays to evaluate receptor activation mechanisms.	Arachidonic acid directly activates the human DP2 receptor, enhancing platelet activation independent of prostaglandin synthesis and involves release of 2-AG.
McIntyre, 2023 [21]	Review/Guidance Commentary	First reported cases of VHD were associated with ergot derivatives acting as 5-HT2B agonists, especially in Parkinson’s disease treatment. These were discontinued due to development of pulmonary hypertension and VHD. Mechanisms involve increased myofibroblast mitogens and deposition of glycosaminoglycans in heart valves. In line with FDA draft guidance, comprehensive assessment of drug-induced VHD risk is recommended. If psychedelics demonstrate efficacy and gain regulatory approval, long-term cardiovascular safety must be monitored using systems like FAERS.
Rouaud et al., 2024 [22]	Review Article	Chronic LSD microdosing may activate 5-HT2B receptors, implicated in VHD. This receptor activation triggers pro-fibrotic pathways: phospholipase C-β, protein kinase C, Src-P, and TGF-β1, along with ERK and MAPK signaling. These cascades can lead to fibroblast activation, cardiac fibrosis, and valvulopathy. Due to structural similarities with drugs known to cause fibrosis (e.g., ergot alkaloids), LSD may carry similar risks. Long-term safety of microdosing remains underexplored, and further clinical investigation is needed
Tagen et al., 2023 [23]	Review Article	No animal or clinical studies were specifically designed to assess VHD risk for psychedelics. Some clinical evidence links MDMA usage with VHD. Chronic psychedelic microdosing is flagged as a potential risk for VHD, but data is insufficient and further studies are necessary to characterize this risk.
Strajhar, 2017 [26]	In vitro experimental study using human blood-derived cells	This study found that LSD modulates innate immune responses by suppressing pro-inflammatory cytokine production (e.g., TNF-α) and enhancing anti-inflammatory cytokines, suggesting immunosuppressive properties through serotonin receptor pathways.
Cloez-Tayarani et al., 2003 [27]	In vitro experimental study using human PBMC	This in vitro study found that serotonin (5-HT) inhibits TNF-α and increases IL-1β production in LPS-stimulated human PBMC, without affecting cytokine mRNA levels. The effects were mediated through 5-HT2A receptors, highlighting serotonin’s modulatory role in immune response.
Nau et al., 2013 [28]	Preclinical experimental study	Activating serotonin 5-HT2A receptors with (R)-DOI potently blocks TNF-α-induced inflammation in both cell cultures and live animals. The anti-inflammatory effects were confirmed to be receptor-specific, suggesting a promising therapeutic avenue for conditions like atherosclerosis and inflammatory bowel disease.
Akasaki and Ohishi, 2020 [33]	Review including case reports of adverse effects of psychedelics.	Describes sympathomimetic effects like tachycardia, hypertension, and hyperthermia of hallucinogens. These substances may also cause vasoconstriction, leading to ischemic or hemorrhagic strokes and acute coronary syndrome. Additionally, they can lower the seizure threshold and contribute to arrhythmias. While fatalities are rare, they have been reported, especially with high doses or polydrug use.
Simonsson et al., 2021 [33]	381,682 responses from NSDUH were analyzed for past one year prevalence of hypertension in lifetime psychedelic users.	Pooled data from the NSDUH (2005–2014) was analyzed to assess prevalence of hypertension in the past one year in lifetime psychedelic users. Data reported that lifetime classic psychedelic use was associated with 14% lower odds of hypertension in the past year, but among the main classes of classic psychedelics, only lifetime tryptamine use was significantly associated with hypertension in the past year, with a 20% lower odds. LSD use did not show significant association with hypertension prevalence in the past one year as suggested by OR of 0.96 (95%CI 0.87-1.05) and p value 0.336.
Ma et al., 2022 [34]	Animal study (mouse model)	Action of SH, a 5-HT 2A receptor antagonist analysed on atherosclerotic plaque formation in aorta of mice models and compared with CDP. SH showed strong anti-atheroscleortic action and synergistic action with CDP due to inhibition of 5-HT pathways.
Strajhar et al., 2016 [35]	Randomized control trial	Serotonin inhibits TNF-α production and increases IL-1β production in LPS-stimulated human PBMC via 5-HT2A receptors. The findings suggest a role for serotonin in modulating inflammation through cytokine regulation.
Holze et al., 2022 [36]	A pooled analysis of four double-blind, randomized, placebo controlled crossover studies including response from 83 volunteers subjected to single dose of 25, 50, 100 and 200 μg LSD.	Acute one-time ingestion of LSD in a controlled clinical setting produced significant changes in heart rate and blood pressure above the doses of 25 μg. Increment in heart rate followed a dose dependent pattern whereas systolic blood pressure > 140, > 160 and > 180 mmHg were observed in 48%, 5% and 0% of all LSD doses, respectively. Tachycardia was observed in 15% of all LSD doses with the maximum heart rate observed being 129 beats/min. Maximum values for diastolic and systolic blood pressure were 103 and 173 mmHg, respectively.
Li et al., 2022 [37]	Review; LSD microdose (50–200 μg)	Microdoses of LSD (50–200 μg) were associated with modest increases in blood pressure and heart rate compared to placebo in healthy subjects. However, these cardiovascular effects were not reported as clinically significant adverse events in the analyzed studies.
Anderson et al., 2019 [38]	278 respondents in three categories: LSD-only (n = 195), psilocybin-only (n = 50), both LSD and psilocybin (n = 33).	Participants were assessed for benefits and challenges associated with microdosing LSD and psilocybin along with frequency of usage of other substances. Data was analyzed using grounded theory analysis and results reported improved mood, anxiety, exercise, reduced caffeine and alcohol intake to establish a taxonomy of benefits of psychedelic use imparting a need for more research to affirm this causality.
Carhart-Harris, 2019 [39]	Review Article	Examined neurobiological mechanisms underlying psychedelic effects. Findings suggest that psychedelics act in the brain through serotonin 2A receptor agonism, leading to increased neuroplasticity, heightened brain entropy, and network disintegration and desegregation. These changes contribute to a relaxation of rigid high-level beliefs, which psychedelic therapy aims to leverage for the revision of maladaptive thought patterns which can be harmful for health including alcohol and tobacco usage.
Teixeira et al., 2022 [40]	Review Article	Explores resurgence of psychedelic research and usage in therapeutic applications of addiction and behaviour changes through mechanism of belief relaxation, increased psychological flexibility and enhanced self-determination motivation. Clinical studies indicate long-term positive behavioral and attitudinal shifts without evidence of disengagement from mainstream society.
de Wit et al., 2022 [41]	Randomized study; 56 healthy participants were assigned to one of three conditions: placebo, 13 mcg LSD, or 26 mcg LSD.	LSD did not significantly impact heart rate or blood pressure in any session.
Oury et al., 2020 [42]	Mechanistic Review Article	Highlights potential cardiovascular risks associated with chronic LSD use, particularly concerning fibrotic remodeling of cardiac tissue. Chronic activation of the 5-HT₂B receptor by substances like LSD can initiate pro-fibrotic signaling cascades involving the activation of phospholipase C-β, protein kinase C, and Src tyrosine kinase, leading to the upregulation of transforming growth factor-β1 (TGF-β1). Subsequently, MAPK pathways, including ERK and p38, are activated, promoting fibroblast proliferation and increased extracellular matrix production. These molecular events contribute to cardiac fibrosis and valvular heart disease.
Raval et al., 2008 [43]	Case Report	Patient developed extensive lower extremity vasospasm after ingesting LSD. The vasospasm was refractory to pharmacologic therapy, necessitating percutaneous transluminal angioplasty for treatment highlighting the potential for LSD to induce severe peripheral vasospasm, leading to significant cardiovascular complications.
Lieberman et al., 1974 [44]	Case Report	Patient experienced fatal intracranial hemorrhage following LSD ingestion. The report raised concerns about the drug's potential to cause acute hypertensive crises or vascular instability, leading to cerebrovascular catastrophes. No preexisting vascular malformation was found on autopsy.
Sobel et al., 1971 [45]	Case Report	A young man developed acute ischemic stroke shortly after LSD use, with angiographic evidence showing vasospasm of the cerebral arteries emphasizing the potential vasoconstrictive effects of LSD leading to reduced cerebral perfusion and stroke in previously healthy individuals.
Aakerøy et al., 2022 [46]	Case Report	A 17-year-old male experienced a tonic-clonic seizure and subsequent cardiorespiratory arrest after ingesting a blotter containing 300 μg of LSD, along with other substances. Despite resuscitation efforts, the patient developed severe neurological sequelae. This case report underscores the potential risks of LSD, especially when used recreationally and in combination with other substances.
[Barnett, 2021]	Cross-sectional survey study. Response rate was 40.20% (106/264); 106 psychiatrists completed the survey out of 264 invited.	It was seen that 80.9% of psychiatrists believed hallucinogens show therapeutic promise for psychiatric conditions, and 60.8% for substance use disorders. A large majority (93.9%) supported further research, and 50.4% expressed intent to incorporate these therapies into practice. Despite growing enthusiasm, there remains a strong need for education on potential adverse effects, especially cardiovascular risks.

With recent advancements like psychedelic-assisted therapies, the medicinal use of LSD and other psychedelics is expected to expand. In this scenario, it becomes even more important for clinicians, cardiologists, and psychiatrists to have knowledge about the potential cardiovascular and immunological adverse effects of LSD. In a survey involving current opinions on psychedelics, the physician participants exhibited strong enthusiasm in learning and improving their knowledge on psychedelic use and assisted therapy. However, the survey also found that the education on potential adverse effects was one of the most desired educational topics on psychedelics, highlighting the importance of residency programs, conference presentations, and continuing medical education meetings to incorporate these topics [47]. Hence, future research into psychedelics should not only address clinical safety and receptor-mediated effects in human models, but also include physician education on side effects, immunological risks, addictive potential, and management of psychedelic-associated ‘bad trips' [48]. Table 2 summarizes the findings of the various studies discussed.

**Table 2:** Summary of the analyses of different studies included in the discussion LSD: lysergic acid diethylamide

Cardiovascular Effect	Description/Mechanism	Acute or Chronic Effect	Impact
Tachycardia (Increased Heart Rate)	Activation of 5-HT2A receptors on the sympathetic system	Acute	Rapid heart rate typically occurs at higher doses, transient.
Increased Blood Pressure (Hypertension)	5-HT2A receptor stimulation causing vasoconstriction and sympathetic activation	Acute	Temporary rise in blood pressure; linked to sympathetic activation.
Platelet Aggregation Inhibition	LSD acts as an antagonist on 5-HT2A receptors in platelets, reducing aggregation	Chronic	Potential protective effect against thrombotic events; may/may not reduce atherosclerosis risk.
Vascular Smooth Muscle Cell Proliferation Inhibition	Antagonism of 5-HT2A receptors in peripheral vascular smooth muscle cells	Chronic	Reduction in smooth muscle cell growth may/may not help reduce cardiovascular events like atherosclerosis.
Reduced Cytokine Release (e.g., TNF-alpha)	5-HT2A receptor action reduces pro-inflammatory cytokine release in immune cells	Chronic	Anti-inflammatory effects may potentially be beneficial in preventing chronic inflammation-related cardiovascular diseases.
Vasospasm (Prolonged)	Rare, due to ergot alkaloid properties, causing prolonged vasoconstriction	Acute	Can lead to peripheral vascular occlusion, major cardiovascular events, and ischemia in susceptible individuals at high doses.
Cardiovascular Collapse	Severe cases in high doses due to prolonged vasospasm, possibly leading to organ failure	Acute	Linked to extreme doses (>100 μg), with serious outcomes like ischemia, stroke, and cardiovascular collapse.
Carotid Artery Occlusion	Resulting from vasospasm due to LSD-induced adrenergic activation	Acute	Risk of cerebrovascular incidents like strokes or neurological deficits in individuals using high doses.
Pro-fibrotic action on cardiac valves and peritoneum	Chronic microdosing of LSD may activate 5-HT2B receptors, triggering pro-fibrotic signaling cascades involving phospholipase C-β, PKC, Src kinase, and TGF-β1. This leads to fibroblast proliferation and extracellular matrix accumulation in cardiac valves and possibly peritoneum.	Chronic/ Acute	Risk of valvular heart disease and cardiac fibrosis with long-term exposure. Clinical risk remains speculative but is concerning in the context of regular microdosing.

## Conclusions

LSD is a lysergamide with agonistic and antagonistic effects in the CNS and the peripheral zones, such as platelets and vascular smooth muscle cells, respectively. LSD triggers a central sympathetic system, which results in cardiovascular system changes. These changes occur due to the stimulation of 5HT2A receptors, leading to tachycardia, high blood pressure, pupillary dilation (mydriasis), and increased body temperature (hyperthermia). LSD, being an antagonist in the peripheral system, causes diminished platelet aggregation, decreased proliferation of vascular smooth muscle cells, and reduced cytokine release from leukocytes. Consequently, a potential decrease in the frequency of atherosclerotic events may be seen.

Various psychedelics possess the property to hamper innate and adaptive immune responses. Declining inflammatory responses and antigen production are usually the common outcomes. To sum up, recreational drugs have both immediate and prolonged impacts, which can lead to cardiovascular dysfunction. Nevertheless, a thorough investigation of the potential advantages and drawbacks of using LSD in a therapeutic context is still required. Clinical trials assessing LSD’s psychological benefits in patients with cardiac conditions will be needed for us to have a better understanding of how LSD can affect pre-existing cardiac conditions, while more detailed epidemiological studies that account for the doses and number of times LSD has been used will be needed to better understand any potential risks to the general population.
